# Advanced quantitative methods in correlating sarcopenic muscle degeneration with lower extremity function biometrics and comorbidities

**DOI:** 10.1371/journal.pone.0193241

**Published:** 2018-03-07

**Authors:** Kyle Edmunds, Magnús Gíslason, Sigurður Sigurðsson, Vilmundur Guðnason, Tamara Harris, Ugo Carraro, Paolo Gargiulo

**Affiliations:** 1 Institute for Biomedical and Neural Engineering, Reykjavík University, Reykjavík, Iceland; 2 Icelandic Heart Association (Hjartavernd), Kópavogur, Iceland; 3 Faculty of Medicine, University of Iceland, Reykjavík, Iceland; 4 Laboratory of Epidemiology and Population Sciences, National Institute on Aging, Bethesda, MD, United States of America; 5 IRRCS Fondazione Ospedale San Camillo, Venezia, Italy; 6 Department of Rehabilitation, Landspítali, Reykjavík, Iceland; Ehime University Graduate School of Medicine, JAPAN

## Abstract

Sarcopenic muscular degeneration has been consistently identified as an independent risk factor for mortality in aging populations. Recent investigations have realized the quantitative potential of computed tomography (CT) image analysis to describe skeletal muscle volume and composition; however, the optimum approach to assessing these data remains debated. Current literature reports average Hounsfield unit (HU) values and/or segmented soft tissue cross-sectional areas to investigate muscle quality. However, standardized methods for CT analyses and their utility as a comorbidity index remain undefined, and no existing studies compare these methods to the assessment of entire radiodensitometric distributions. The primary aim of this study was to present a comparison of nonlinear trimodal regression analysis (NTRA) parameters of entire radiodensitometric muscle distributions against extant CT metrics and their correlation with lower extremity function (LEF) biometrics (normal/fast gait speed, timed up-and-go, and isometric leg strength) and biochemical and nutritional parameters, such as total solubilized cholesterol (SCHOL) and body mass index (BMI). Data were obtained from 3,162 subjects, aged 66–96 years, from the population-based AGES-Reykjavik Study. 1-D k-means clustering was employed to discretize each biometric and comorbidity dataset into twelve subpopulations, in accordance with Sturges’ Formula for Class Selection. Dataset linear regressions were performed against eleven NTRA distribution parameters and standard CT analyses (fat/muscle cross-sectional area and average HU value). Parameters from NTRA and CT standards were analogously assembled by age and sex. Analysis of specific NTRA parameters with standard CT results showed linear correlation coefficients greater than 0.85, but multiple regression analysis of correlative NTRA parameters yielded a correlation coefficient of 0.99 (P<0.005). These results highlight the specificities of each muscle quality metric to LEF biometrics, SCHOL, and BMI, and particularly highlight the value of the connective tissue regime in this regard.

## Introduction

The progressive degeneration of aging muscle, known as Sarcopenia, has been consistently identified as an independent risk factor for mortality [1–7], and while its prevalence has been characterized by profound decreases in both physical activity and vitality, a normative quantitative definition for its diagnosis and etiology remains debated around the world [8–10]. Nonetheless, extant clinical literature has interpreted its impact on physiological function and cite Sarcopenia as the primary loss for muscle strength [11–13]. However, the degree to which this loss of muscle strength may be attributed to the loss of muscle mass remains uncertain. Current literature additionally cites the loss of muscle quality as a significant dictator for the loss of muscle function–especially when in conjunction with a loss of muscle mass [14–16]. Nonetheless, methodological comparisons for the precise, non-invasive quantification of the progressive reduction of muscle quality remain disparately described in literature. Standardizing a quantitative methodology for myological assessment in this regard would allow for the generalizability of sarcopenia research to the indication of compensatory targets for clinical intervention.

### Skeletal muscle and aging

Aging skeletal muscle has a significantly reduced proportion of glycolytic type II muscle fibers compared to young muscle, which in-turn directly elicits diminished myofibril contractility [17, 18]. Additionally, aging skeletal myofibers significantly lack the ability to process triglycerides, resulting in increased lipid droplet storage along myocyte cell membranes [19]. This increased adiposity and decreased contractility has been linked to mitochondrial dysfunction and impaired oxidative metabolism, which has been shown to secondarily induce metabolic insulin resistance and Type 2 diabetes mellitus in patients [20, 21]. In general, non-contractile tissue infiltration, in accordance with a loss of muscle mass, confers an increased risk for frailty, disability, reduced mobility, and eventual hospitalization [22]. Indeed, a direct link between aging and increasing degrees of non-contractile tissue infiltration in thigh muscle has been observed [23, 24].

Studying how these changes affect mobility is the prime motive for lower extremity function (LEF) research, which cites LEF as the main indicator for mobility as a clinical screening tool [25]. LEF is generally assessed by measuring walking capacity (gait speed) and leg strength [26]. Altogether, the association of sarcopenic muscle degeneration with decreasing LEF illustrates how aging induces mobility impairment, incident disability, and eventual mortality [27–30].

### The role of imaging: Muscle quality as a clinical comorbidity index

Muscle biopsy is the standard clinical procedure used for the assessment of muscle, but the procedure is invasive and occasionally limited in relevance by the small size of excised tissue. However, recent investigations have realized the potential of X-ray computed Tomography (CT) and Magnetic Resonance Imaging (MRI) to describe muscle quality and composition. This is often performed either quasai-quantitatively, via the visual grading of muscle structure morphologies [31–34], or quantitatively via the computation of muscle cross-sectional areas and radiodensitometric absorption values in CT, measured in Hounsfield units (HU) [35–40]. Despite the superior soft tissue contrast in MRI and non-dependence on the use of ionizing radiation, CT has higher spatial resolution and is comparatively less obfuscated by technical variations in machine preparation and acquisition protocols [41, 42]. These notions are critical when attempting to discern diagnostically-relevant information from cross-sectional images of soft tissue.

While the propensity of CT imaging literature describing sarcopenia differs largely regarding analytical methodology, one metric that remains ubiquitous is the use of average HU values to characterize muscle quality. Goodpaster et al report the use of average HU values for quantifying skeletal muscle lipid content, highlighting the potential for the method to investigate the association between muscle composition and function [43–45]. Hicks et al likewise utilized average HU values within lower back and posterior muscle groups to illustrate the inverse relationship between these muscle qualities and the prevalence of lower back pain [46]. Additionally, Sur et al utilized average HU values weighted by total muscle area to show a correlation between muscle quality in the psoas and serious post- Pancreaticoduodenectomy complications [47]. Likewise, Lang et al utilized average HU attenuation values within thigh muscles to predict the propensity for hip fracture in elderly, otherwise healthy subjects [48].

While average HU values might indeed describe general shifts in adiposity, generalizing CT image matrices in this regard risks eliminating other distribution characteristics that could explain additional subtle changes in muscle properties. We have previously shown the potential for computational modelling of entire radiodensitometric distributions using the novel nonlinear trimodal regression analysis (NTRA) method [49]. The eleven unique regression model parameters inherent to the NTRA method have shown utility in characterizing muscle HU distributions in small cohorts, but using a large subject dataset to compare its utility to those of current standard analyses (cross-sectional area and average HU value) has yet to be reported. This was the prime motive for the present study, wherein CT cross-sections from the midthighs of 3,162 community-dwelling subjects from ages 66–96 were assessed to correlate upper leg muscle quality with continuous class LEF biometrics as well as biochemical and nutritional parameters, such as total solubilized cholesterol (SCHOL) and body mass index (BMI). The aim for this investigation was twofold: 1) to further develop an understanding of quantitative muscle quality assessment as a potential subject-specific modeling tool, and 2) to correlate NTRA parameters and parameters obtained using standard CT analyses with lower extremity functions and biochemical parameters.

## Materials and methods

### Subject recruitment

The 3,162 volunteer subjects for this study were recruited from participants in the prospective population-based five-year follow-up investigation of the Age Gene/Environment Susceptibility Study in Reykjavík, Iceland (AGESII-Reykjavik) [50–53]. The follow-up occurred from 2007–2011 and the percent of original subjects who participated was 71%. Written and informed consent was obtained from all participants [50–53]. Ethical approval for patient data acquisition was obtained by the Icelandic National Bioethics Committee, which serves as the Institutional Review Board for the Icelandic Heart Association (RU Code of Ethics, cf. Paragraph 3 in Article 2 of the Higher Education Institution Act no. 63/2006).

The follow-up group initially included 3,316 subjects whereof 3,169 participated in the acquisition of CT scans of the mid-thigh. Seven additional subjects were excluded due to their non-participation in biometric or comorbidity measurements. The remaining 3,162 subjects (1,327 males, 1,835 females, mean age 79.9±4.8) participated in least one of the investigation’s data measurements, and, out of this total investigation cohort, the percentage of participants in each measurement ranged from 93.9% to 98.8%. Only those whose data mas acquired for each metric were included into the present work.

### CT acquisition and segmentation

All participants in the project were scanned with a 4-row CT detector system at 120-kV (Sensation; Siemens Medical Systems, Erlangen, Germany) as previously described [50–53]. The localized scanning region extended from the iliac crest to the knee joints. For each subject, a single 10-mm thick transaxial mid-femur section was utilized in generating HU distributions and calculating fat and muscle cross-sectional areas. Prior to the transaxial imaging, the correct position for mid-femur imaging was determined by measuring the maximum length of the femur on an anterior-posterior localizer image, followed by locating the center of the femoral long axis.

Fat and muscle lean areas were segmented using the fascial plane outline between muscle and subcutaneous fat as previously described [49]. A manual contouring program to draw the contours of the total muscle bundle, and a threshold was chosen within each region to select voxels with CT densities greater than the maximal density of fat, as documented [49, 51]. The lean area of each muscle region was then calculated as the number of voxels above this threshold, and lean tissue attenuation was defined as the mean CT density of these thresholded voxels.

### LEF biometrics and SCHOL/BMI measurements

LEF biometrics were assessed as part of the aforementioned AGES-Reykjavik Study. Continuous-class measurements that were available for investigation included normal and fastest-comfortable gait speed (GSN and GSF, respectively), timed up-and-go (TUG), and isometric leg strength (STR) [54]. Gait speeds were calculated in seconds over a distance of six meters, and two trials were averaged for each subject according to published protocol [55]. The TUG test measured the time taken to stand from a seated position, walk three meters, turn around, walk back to the chair, and sit down; TUG is a well-reported screening metric for assessing balance problems and daily activity declination [56]. Finally, STR was measured via knee extension using an adjustable digital dynamometer on a fixed chair (Good Strength, Metitur, Palokka. Finland). STR was measured at a fixed knee angle of 60 degrees from full extension, and the subject’s ankle was fastened to a strain-gauge transducer. STR was measured by taking the greater measured force of two knee extensions; each trial was four seconds in duration, with a 30 second rest between trials.

The SCHOL and BMI parameters from the AGES-Reykjavik study were included on the basis of both their availability as age-related comorbidities and their being continuous-class in nature. BMI was calculated as the subject’s weight (kilograms) divided by height (in meters) squared, as previously reported [57]. Fasting total solubilized cholesterol levels were measured via a Hitachi 912 with comparable enzymatic procedures (Roche Diagnostics, Mannheim, Germany) [58]. All body weight and lipid measurements fulfilled the criteria of the National Institute of Health and the National Cholesterol Education Program for accuracy and precision.

### Pixel distribution binning and smoothing

For each subject, HU distributions were derived from summing and transforming each pixel’s CT number value according to the following linear transformation expression:

HU=CT×2.26625−190 (1)

Following transformation, HU values were binned into 128 bins, as typical for CT assessment protocols [59]. Resultant histograms were smoothed by a non-parametric fitting algorithm to obtain underlying empirical probability density functions (PDF) for each histogram. Each PDF was then exported for NTRA regression analyses.

### Nonlinear trimodal regression analysis (NTRA) method

The method utilized to computationally define each HU distribution was a modified methodology for nonlinear regression analysis. First, the general equation for each distribution was defined as a quasi-probability density function by summing two skewed and one standard (α = 0) Gaussian distributions:

$$\sum_{i=1}^{3}\varphi \left(x,N_{i},\mu_{i},\sigma_{i},\alpha_{i}\right)=\sum_{i=1}^{3}\frac{N_{i}}{\sigma_{i}\sqrt{2\pi }}e^{-\frac{{\left(x-\mu_{i}\right)}^{2}}{2\sigma_{i}{}^{2}}}erfc\left(\frac{\alpha_{i}\left(x-\mu_{i}\right)}{\sigma_{i}\sqrt{2}}\right)$$ (2)

where ***N*** is the amplitude, ***μ*** is the location, ***σ*** is the width, and ***α*** is the skewness of each distribution–all of which are iteratively evaluated at each CT bin, ***x***. This definition is resultant from the hypothesis that each HU distribution is trimodal, in that they consist of three separate tissue types whose linear attenuation coefficients occupy distinct HU domains: namely, fat (i = 1) [-200 to -10 HU], loose connective tissue and atrophic muscle (i = 2) [-9 to 40], and normal muscle (i = 3) [41 to 200]. Additionally, we hypothesized that the inwardly-sloping asymmetries within the fat and muscle peaks could be described by skewnesses of their probability density functions, whereas the central connective tissue distribution was assumed to be a normal, non-skewed Gaussian distribution. Utilizing this definition, a theoretical curve was generated by employing a iterative generalized reduced gradient algorithm via minimization of the sum of standard errors at each CT bin value, ***x***, thereby generating an 11-parameter matrix of PDF variables. This algorithm iterates each function variable according to the computed variance of each step, and the selection of new trial values is guided by computing the rates of change of this variance as new inputs are generated. The minimization of the sum of standard errors at each point, and thereby the maximization of R^2^, was computed according to their standard definitions.

**Fig 1:**
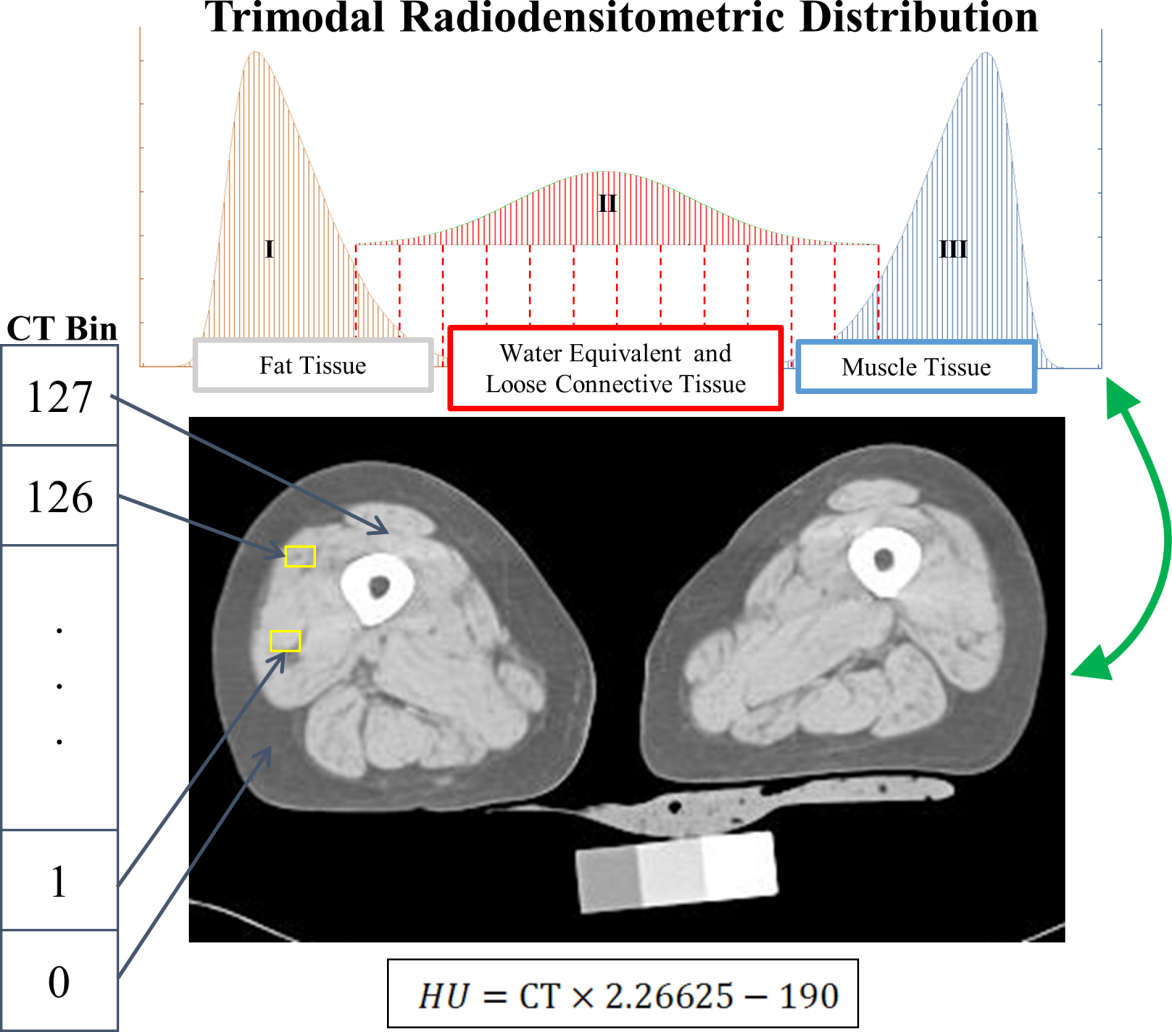
Diagram depicting the three components of the trimodal radiodensitometric distribution utilized in this study. This figure illustrates the location and skewnesses of each PDF, with tissue types as follows: I) Fat [-200 to -10 HU], II) Water Equivalent and Loose Connective Tissue [-9 to 40 HU], and III) Muscle [41 to 200 HU].

An illustration of the results of this concept is shown in Fig 1, where each of the three tissue types and their respective PDFs have been depicted.

### 1-D K-means clustering discretization of LEF biometrics and SCHOL/BMI

With the modern adoption of electronic medical records (EMRs), the availability and breadth of clinical data for use in medical research has markedly increased [60]. Commensurately, there has been a rising interest in the development of novel algorithms from the fields of data mining and machine learning for the processing of medical data [61, 62]. One of the common preprocessing steps utilized prior to the employment of many of these algorithms is the discretization of continuous variables. Discretization eliminates the necessity for presumptions regarding distribution characteristics, as the method employs the counts within the dataset to directly evaluate conditional probabilities [63].

For this reason, the present work identified the discretization of continuous class LEF biometrics, SCHOL, and BMI as a strategic priority. Unsupervised methods for discretization were initially selected, as they eliminate the requirement for class labels and can eventually be utilized for multiple applications, unlike supervised discretization methods [63, 64]. After investigation, the transformation of each continuous class variable was ultimately performed using one-dimensional (1-D) k-means clustering [65]. In this method, each cluster-derived bin had observations sorted using a medoid-partitioning algorithm [66]. The number of groups (k) was calculated using Sturges’ formula for class selection, which implicitly bases bin sizes upon the range of the dataset, assuming a normal distribution of class values [63, 67]:

$$k={\text{log}}_{2}n+1$$ (3)

where the brackets denote the ceiling function, n is the dataset population, and k is the number of bins. This yielded a k of twelve for each LEF and comorbidity parameter the study. Finally, to assess the fidelity of linear relationships between muscle quality CT standards and NTRA parameters, subjects were discretized by age and sex. Discretization was again unsupervised, but this time by equal frequency due to the truncated nature of age, which is, in concept, a continuous class variable [68]. Using Sturges’ formula here resulted in 11.4 bins for men and 11.8 for women, so twelve bins were chosen for the sake of simplicity in assessing comparative linear regressions. This yielded bin populations of 111 for men, and 153 for women.

### Statistical analyses

Linear regression models were utilized to statistically correlate the relationships of each independent variable (LEF biometrics, SCHOL, and BMI) with respect to dependent variables (NTRA parameters and standard CT analyses). Multiple regression models were then utilized on assemblies of each dependent variable whose correlation coefficients described greater than 85% of the variance in each independent metric. One-way ANOVA and F-tests for overall significance were compiled from each multiple regression model to illustrate statistically meaningful correlation.

## Results and discussion

### Standard CT analyses on LEF biometrics and SCHOL/BMI

**Fig 2:**
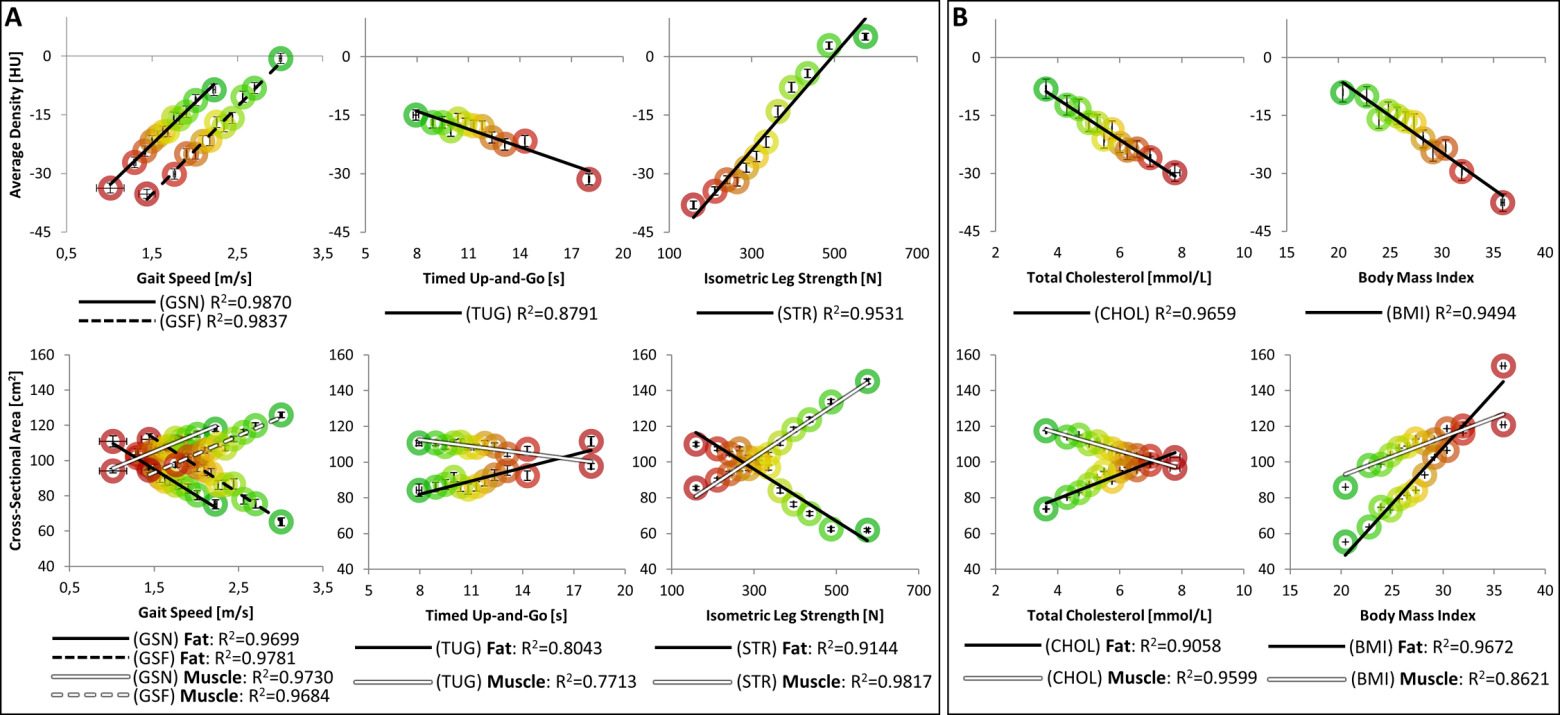
Current CT standard analyses against A) LEF biometrics and B) SCHOL and BMI. Note that each population bin is shown with colored circles ranging from the unhealthiest (red) to the healthiest group (green), as defined by each parameter. Regression lines are likewise shown for each data series, along with their respective coefficients of determination (R^2^).

Fig 2 depicts the results from standard CT analyses on LEF biometrics, SCHOL, and BMI. Each population bin is shown with colored circles ranging from the most unhealthy comorbidity measurement or LEF value (red) to the healthiest group (green). Regression lines are likewise shown for each data series, along with their respective coefficients of determination (R^2^).

As is evident in Fig 2, the assessment of LEF biometrics, SCHOL, and BMI with current CT standard methods yielded a host of physiologically evident results that are, to our knowledge, presented in this manner for the first time. Firstly, fat cross-sectional areas held an inverse relationship with normal gait speeds, fast gait speeds, and isometric leg strengths, while conversely showing positive correlation with timed up-and-go speeds (although while, in general, capturing considerably less of the parameter variance: 0.80 and 0.77 for fat and muscle, respectively). At the same time, muscle cross-sectional areas showed a directly inverse relationship with LEF biometrics, compared to fat. Likewise, average HU values show commensurately expected trends: the worse the LEF performance, the greater the shift in average soft tissue density towards fat (increasingly negative HU values). While these relationships are wholly explainable in terms of muscle physiology, the apparent trends evidenced by SCHOL and BMI are more intriguing: as any significant relationships without the removal of potentially-obfuscating, but entirely relevant patient details might not have been expected. In this regard, it can be seen how increasingly unhealthy BMI and SCHOL resulted in increasing fat areas and greater shifts towards negative average HU values. With increasing BMI, both fat and muscle areas increased together, although the rate of increase in fat was greater than the increase in muscle area. It is apparent that most current standard metrics held linear regression coefficients of determination greater than 0.85, suggesting their general utility in capturing the population variance of each metric.

### NTRA analyses on LEF biometrics and SCHOL/BMI

**Fig 3:**
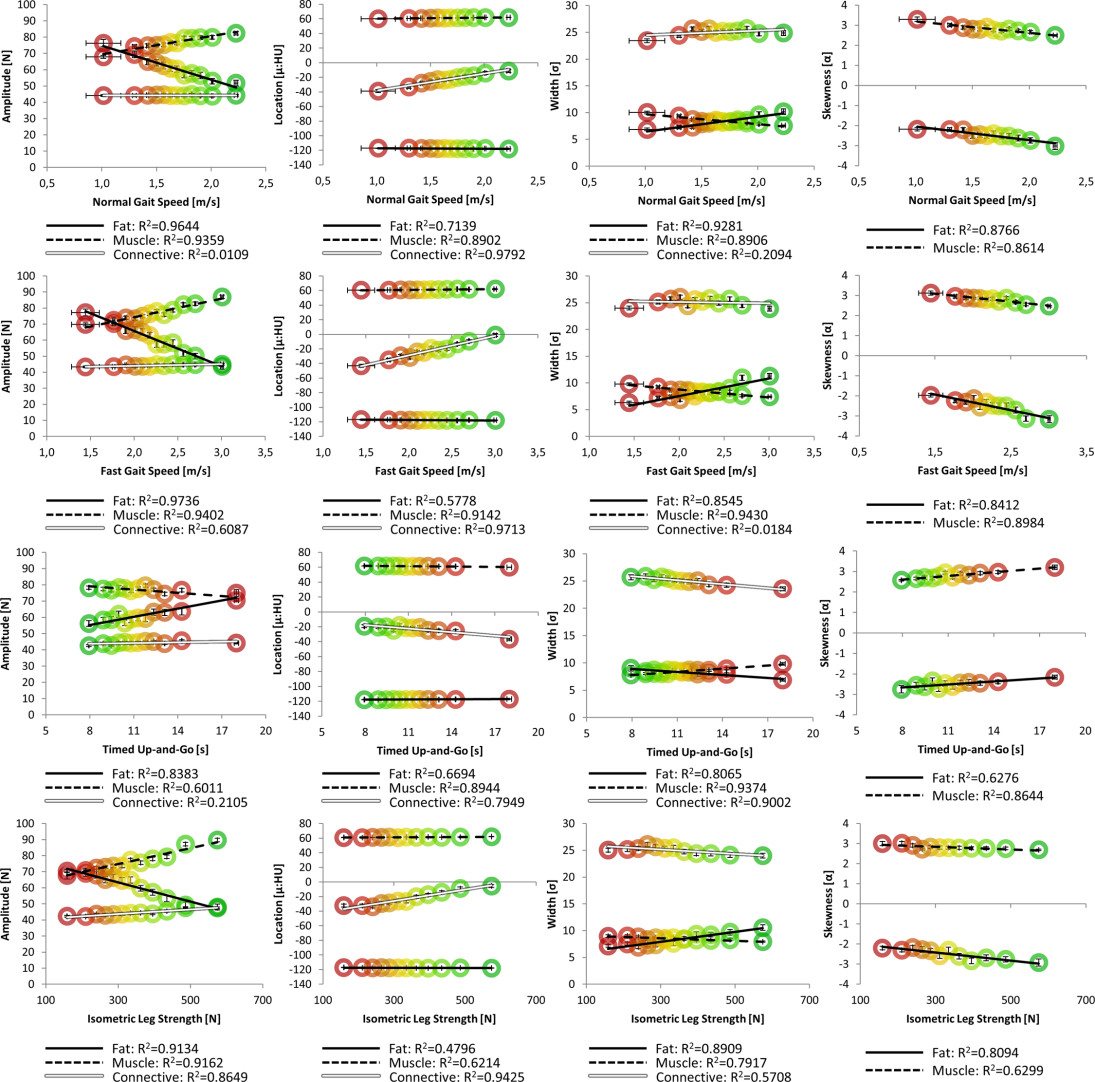
NTRA parameters against LEF biometrics. Each population bin is shown with colored circles ranging from the unhealthiest LEF value (red) to the healthiest group (green). Regression lines and their corresponding R^2^ values are analogously shown for each data series.

**Fig 4:**
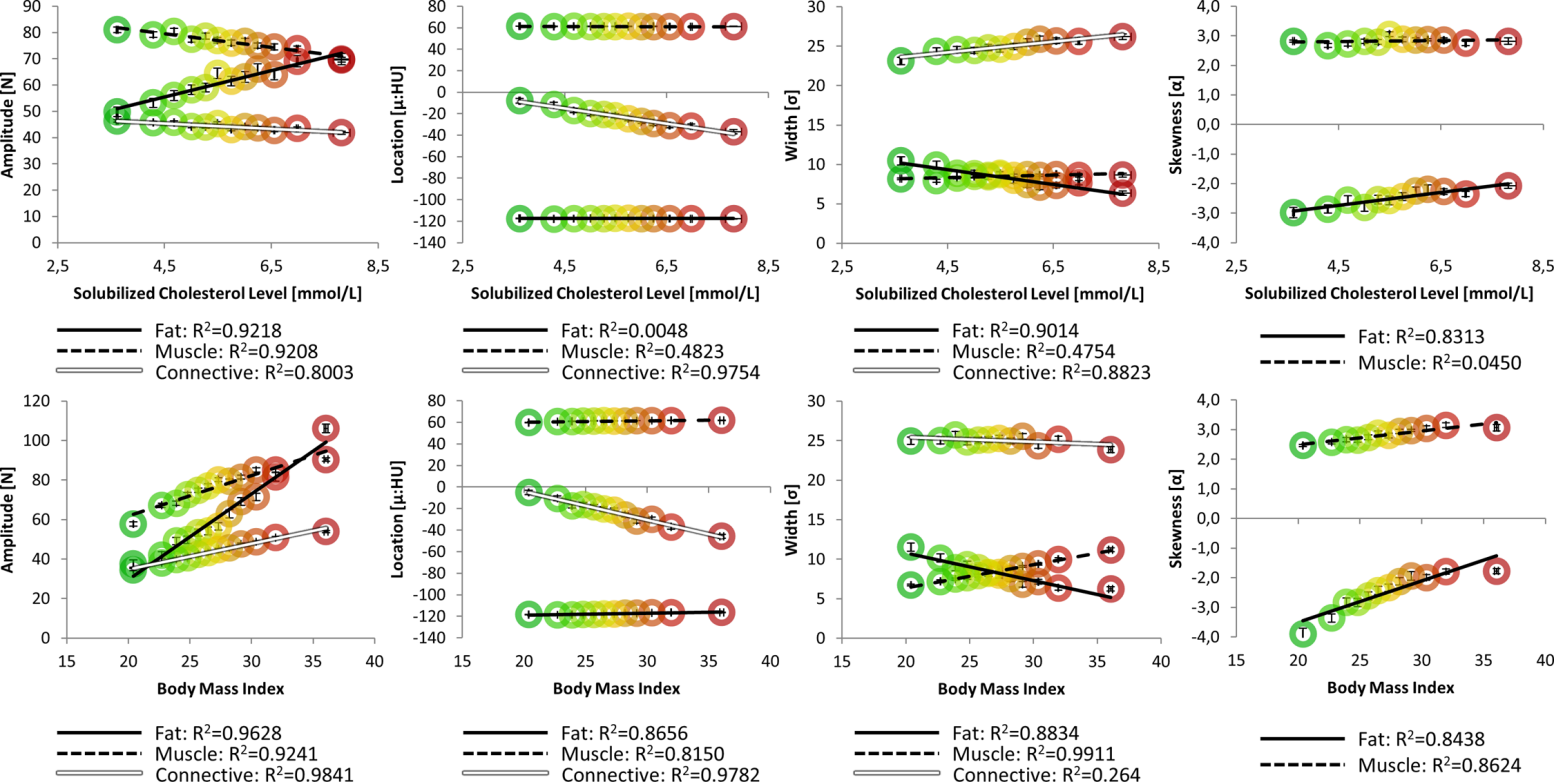
NTRA parameters against SCHOL and BMI. Population bins are shown with colored circles ranging from the unhealthiest measurement (red) to the healthiest group (green), along with their regression lines and R^2^ values.

As a complimentary analysis to standard CT methods, NTRA parameters were analogously assembled. Figs 3 and 4 depict linear regressions of distribution amplitudes, locations, widths, and skewnesses against LEF biometrics and SCHOL/BMI, respectively.

What is first apparent from Fig 3 is the great variance in specificity among coefficients of determination. Many parameters yield strongly linear relationships on the order of or greater than standard CT analyses (R^2^>0.85), while others yield minimal or no correlation. It may be readily hypothesized that the amplitude parameter varies identically with cross-sectional area against these metrics, assumptively suggesting a direct relationship to the overall quantity of each soft tissue type. Indeed, this is readily apparent in fat and muscle amplitudes: increasing fat amplitudes with decreasing LEF performance can be seen, along with an inverse muscle amplitude relationship. What is clearly different is the inclusion of the loose connective/water equivalent tissue regime, which according to these results seems less tied to quantity, and instead more to overall tissue quality–with the clear exception of isometric leg strength yielding a strong relationship with connective tissue amplitude. For instance, it is apparent here how connective tissue locations clearly shift towards more negative HU values with decreasing LEF performance. In the context of the aforementioned presence of myosteatosis in aging muscle, the commensurate presence of fibrosis has been observed [69, 70], but its precise measurement by standard CT metrics remains impossible. Pushing further into other medical imaging modalities such as MRI or ultrasound, to a degree, severe tissue fibrosis is detectable using traditional image processing modalities; however, as their employment methods are generally more tuned to detect and diagnose pathological hepatic fibrosis, they may not be optimized for detecting the comparatively-minute changes associated with the onset of sarcopenia. Indeed, the contribution of fibrosis to traditional metrics of muscle quality remains an essential target for further investigation; it is thereby enticing to observe how the inclusion of the present loose connective/water equivalent tissue regime in NTRA analysis may serve as a direct metric for fibrosis with further study.

It is likewise possible to hypothesize that the NTRA width parameter might be directly related to the overall variation in pixel values for each tissue regime, as an increasing range of HU values would simply imply an increasing Gaussian distribution width. However, these data suggest that this parameter is minimally related to gait speed and isometric leg strength, but is instead almost singularly tied to timed up-and-go duration, as decreasing width clearly confers longer TUG times. In all LEF metrics, however, the same relationship is observed: increasing fat width and decreasing muscle width confer decreasing LEF performance. This could likewise be more related to notions of tissue quality, as myosteatosis would present itself as an increase in partial volume effect from an increase in the relative proximity of inter- and intramuscular fat and lean muscle. However, why this may be singularly apparent in the fat distribution remains unclear.

In regards to the NTRA location parameter, as before, our results indicate that shifts in these values are less related to muscle and fat, but highly connected to loose connective/water equivalent tissue. The standard CT analogue, in the case of location values would be the average HU value. When comparing the linear relationships of these parameters, analogous shifts towards increasingly negative values are seen, in accordance with decreasing LEF performance.

Finally, the NTRA skewness parameter may again be potentially thought of as a direct descriptor for muscle quality, as intermuscular adiposity may incur the migration of lean tissue pixels towards the center of the overall distribution, resulting in an increasingly-skewed PDF shape. Intriguingly, all LEF parameters present the same trend: increasing muscle skewness and less-negative fat skewness values inversely correlate with LEF performance. However, this dependency is more tied to muscle than fat–an explanation for which, again, remains unclear.

**Fig 5:**
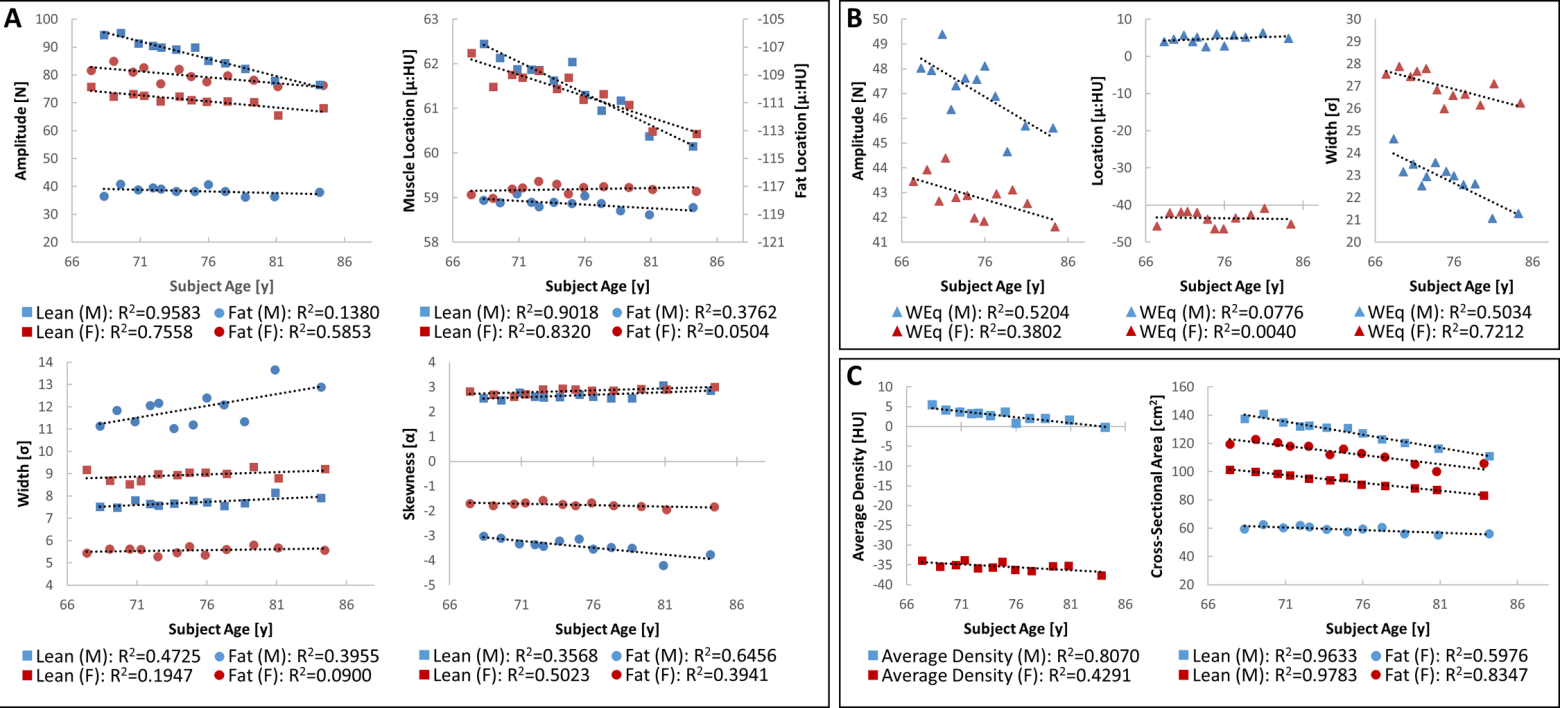
Assembly of NTRA parameters with high correlation fidelity. Parameters were selected for yielding LEF and SCHOL/BMI correlation coefficients greater than 0.85.

Finally, Fig 4 depicts linear relationships with NTRA parameters in accordance with SCHOL and BMI values. Here, one can immediately note analogous dependencies, as compared to LEF performance biometrics. Indeed, increasing fat amplitudes and decreasing muscle amplitudes confer healthier measurements for BMI and SCHOL levels. Shift in connective tissue location confer increasingly negative values, in conjunction with greater BMI and higher SCHOL levels. Increasing fat widths and decreasing muscle widths analogously indicate increasingly unhealthy measurements; intriguingly, higher SCHOL values were seen with increasing connective widths. Increases in muscle skewnesses and less-negative fat skewnesses correlated with less healthy BMI and SCHOL values. To summarize these results, Fig 5 depicts the specificities of each NTRA parameter, showing associated LEF biometrics and SCHOL/BMI values whose linear regressions yielded coefficients of determination of at least 0.85.

Multiple regression analyses were performed on all standard CT analyses and NTRA parameters with individual linear regression coefficients of determination over 0.85 for each LEF and SCHOL/BMI metric. Results from these analyses produced multiple regression models involving each parameter, and all models yielded multiple correlation coefficients greater than 0.99 with ANOVA and F-test results with P<0.005. These results indicate the high fidelity of multivariate correlation and robust statistical significance when all highly-linear LEF biometrics and SCHOL/BMI values are included. Additionally, such strong multivariate correlation suggests, at the very least, that these NTRA parameters should be included alongside extant standard methods for muscle quality quantification.

### NTRA and standard CT analyses on age and sex

**Fig 6:**
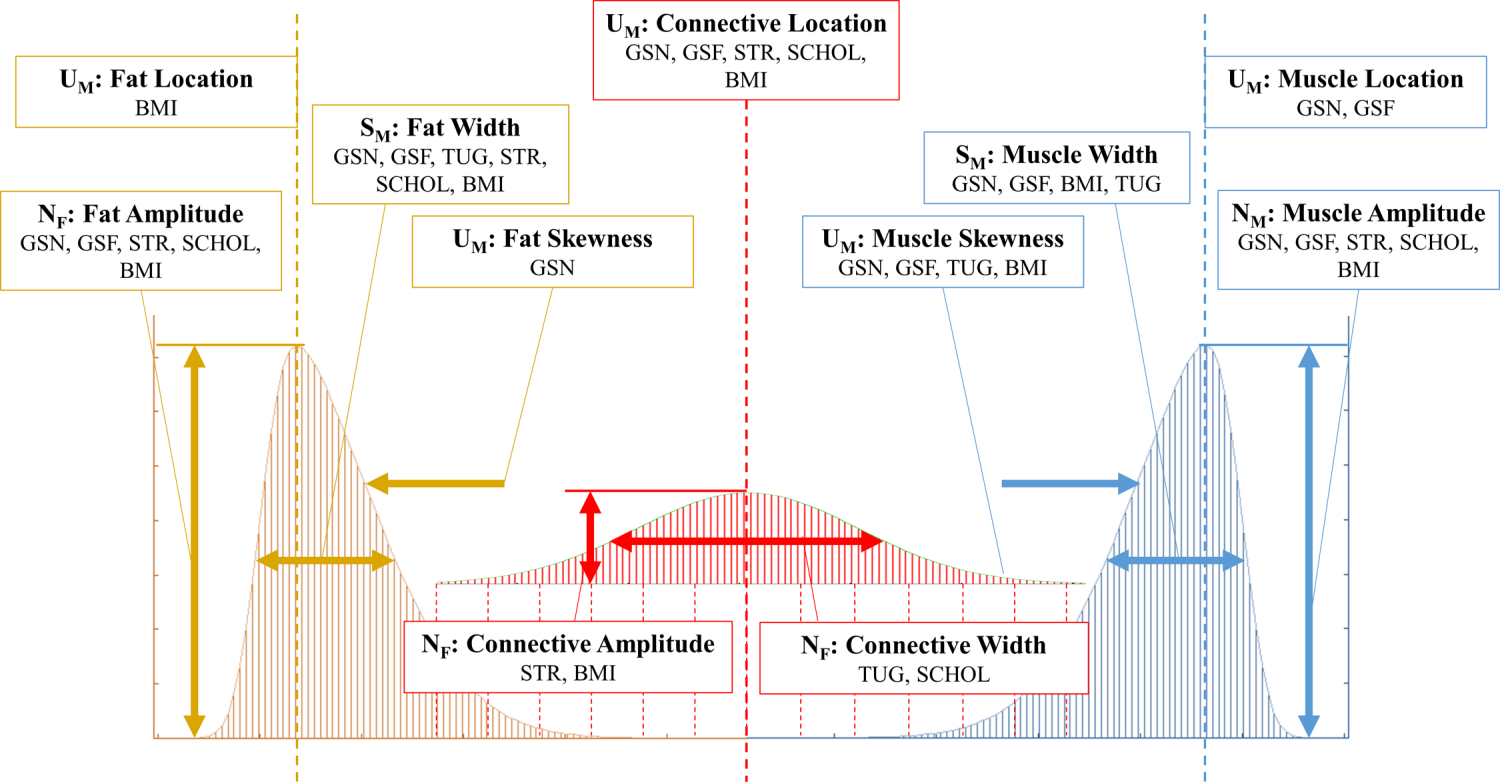
NTRA and standard CT analyses against age and sex. Each age bin depicts the differences between male (blue) and female (red) subjects. A) Shows each NTRA parameter against subject age for fat (circles) and muscle (squares). In addition, B) shows NTRA parameters associated with loose connective tissue, and C) shows standard CT analyses. Regression lines and their respective coefficients of determination are depicted here for each data series.

Fig 6 depicts the results from NTRA and standard CT analyses on age and sex.

As an additional investigation, NTRA parameters and standard CT metrics were assessed for linear correlation with age, stratified by subject sex. Fig 6 illustrates these results. What is immediately evident is that age and sex may be possible confounding factors in muscle quality analysis. However, while the linear dependencies of NTRA and standard CT analysis metrics are clear in some cases (analogously greater than 0.85), correlation coefficients are overall much lower than those of LEF biometrics and SCHOL/BMI parameters. This altogether suggests the necessity for correcting our model for age and sex, but likewise underscores the comparative ineffectuality of classic clinical assumptions of sarcopenic muscle degeneration based on age and sex. Nonetheless, modeling muscle degeneration as shown without such correction yet with high degrees of statistical significance is illuminating for further discussion, as being able to predict changes in LEF biometrics and SCHOL/BMI values implicated in sarcopenia regardless of age and sex may be of considerable inherent value.

### Study strengths and limitations

As previously mentioned, partial volume effects can affect HU distributions, which can lead to errors in parameter estimation, but such errors would be systematic throughout the whole dataset and therefore not affect the overall results. The strength of this study is the large patient cohort evaluated and the trends that show high correlation with the lower extremity functions as well as the biochemical and nutritional parameters. However, since age and sex may be possible confounding factors in muscle quality analysis due to their occasionally-strong linear dependencies on NTRA and standard CT analysis metrics. While this suggests the necessity for model correction in this regard, age and sex correlation coefficients were much lower overall compared to those of LEF biometrics and SCHOL/BMI parameters. It would be a valuable future step in the direction of this work to rigorously evaluate these potential dependencies. Furthermore, as is true in any such study, the use of more subjects and aging comorbidities will be essential to reinforcing any of the physiological interpretations reported here, and further discussion regarding potential applications and adjustments to the reported model will be requisite.

## Conclusions

The present study illustrates the notion that rigorous quantification of entire HU distributions can elicit many unique assessment parameters and therein provide additional information regarding muscle quality alongside extant current standard CT analysis methods. Parameters obtained by the NTRA method demonstrated higher correlation with lower extremity functions and biochemical variables than parameters obtained from standardized CT analysis. These results highlight the methodological importance of including entire radiodensitometric distributions in accordance with the NTRA assessment method and provide further insight into how muscle degeneration in sarcopenia can be optimally diagnosed and quantified.
